# Stabilization of a soluble, native-like trimeric form of an efficiently cleaved Indian HIV-1 clade C envelope glycoprotein

**DOI:** 10.1074/jbc.M117.776419

**Published:** 2017-03-10

**Authors:** Shubbir Ahmed, Tripti Shrivastava, Naresh Kumar, Gabriel Ozorowski, Andrew B. Ward, Bimal K. Chakrabarti

**Affiliations:** From the ‡ HIV Vaccine Translational Research Laboratory, Translational Health Science & Technology Institute, NCR Biotech Science Cluster, Faridabad, Haryana 121001, India,; the § Department of Integrative Structural and Computational Biology, Center for HIV/AIDS Vaccine Immunology and Immunogen Discovery, International AIDS Vaccine Initiative Neutralizing Antibody Center and Collaboration for AIDS Vaccine Discovery The Scripps Research Institute, La Jolla, California 92037, and; the ¶ International AIDS Vaccine Initiative, New York, New York 10004

**Keywords:** AIDS, human immunodeficiency virus (HIV), protein expression, protein purification, vaccine, vaccine development, viral protein

## Abstract

Designing an effective HIV-1 envelope glycoprotein (Env) immunogen for elicitation of broadly neutralizing antibodies (bNAbs) is a challenging task because of the high sequence diversity, heavy glycosylation, and inherent meta-stability of Env. Based on the antigenic profile of recently isolated bNAbs, the rational approach to immunogen design is to make a stable version of the Env trimer, which mimics the native trimeric Env present on the viral surface. The SOSIP.664 form of a clade A Env, BG505, yields a homogeneous and well ordered prefusion trimeric form, which maintains structural integrity and desired antigenicity. Following the same approach, we attempted to stabilize a naturally occurring efficiently cleaved clade C Env, namely 4-2.J41, isolated from an Indian patient. Although the SOSIP form of 4-2.J41 failed to produce reasonably well ordered trimers, the 4-2.J41.SOSIP.664 Env could be stabilized in a native-like trimeric form by swapping a domain from BG505 Env to 4-2.J41 Env. Using various biochemical and biophysical means we confirmed that this engineered Env is cleaved, trimeric, and it retains its native-like quaternary conformation exposing mostly broadly neutralizing epitopes. Moreover, introduction of a disulfide bond in the bridging sheet region further stabilized the closed conformation of the Env. Thus, our 4-2.J41.SOSIP.664 Env adds to the increasing pool of potential immunogens for a HIV-1 vaccine, particularly for clade C, which is the most prevalent in India and many other countries. Besides, the approach used to stabilize the 4-2.J41 Env may be used successfully with Envs from other HIV-1 strains as well. Additionally, a soluble native trimeric form of an efficiently cleaved membrane-bound Env, 4-2.J41, may be beneficial for immunization studies using various prime-boost strategies.

## Introduction

HIV-1 Env^3^ is a trimer of heterodimers of gp120 and gp41 that are associated non-covalently after cleavage of gp160 by an endogenous protease (1). The trimeric Env on the HIV-1 surface facilitates viral entry into the host cell through receptors and is the target of neutralizing antibodies (2).

In some rare subjects, broadly neutralizing antibodies (bNAbs) are spontaneously generated about one to several years post-infection. An immunological tussle between escape mutants and affinity maturation yields a favorable path leading to gradual production of bNAbs (3). These bNAbs generally target well conserved epitopes on the Env surface (4) and block viral entry into the host cells (5, 6). In recent years, detection and isolation of several bNAbs from such infected individuals (7, 8) has raised hope that it may be possible to stimulate the immune system to elicit bNAbs by vaccination with appropriate immunogens.

The critical epitopes on Env, which are recognized/targeted by bNAbs are conformational in nature and highly dependent on quaternary packing of the Env (9). Therefore, to elicit neutralizing antibody response, the rational approach is to design Env immunogens, which can present its native-like conformation to the host immune system. However, designing an Env immunogen, which is in a soluble, native-like prefusion state and selectively displays neutralizing determinants while occluding the epitopes targeted by non-neutralizing antibodies (non-NAbs), is challenging, due to the enormous sequence diversity and an intrinsically metastable nature of the Env (10). The regional prevalence of different subtypes further worsens the prospect of designing a single universal vaccine for all subtypes. Growing evidences support the use of multiclade immunization approaches (11). Thus, to overcome HIV-1 diversity, there is a need to increase the repertoire of Env-based immunogens across different subtypes by designing clade-specific immunogens based on regional prevalence of a particular subtype for structural and immunogenicity studies (12).

Currently multipronged approaches are being pursued to design HIV-1 Env immunogens. A well documented strategy to enhance expression, decrease aggregation, and increase structural homogeneity is to truncate the *env* sequence before the membrane proximal external region at amino acid 664 and introduce six arginine (6R) residues at the furin cleavage site (REKR). A disulfide linkage between residue 501 of gp120 and 605 of gp41 (SOS) stabilizes the trimeric conformation, whereas the isoleucine to proline substitution (I559P) in the heptad repeat 1 (HR1) region stabilizes the Env in its prefusion state (13). Envs expressed from this construct are known as SOSIP.664 Envs. Other strategies include adding a foldon-like trimeric motif at the C terminus of gp41 (14) or inserting a peptide linker between gp120 and gp41 (15, 16). Among the several Envs of different subtypes, which have been stabilized by using the strategies mentioned above, the soluble, native-like trimeric clade A Env, BG505.SOSIP.664, is the best characterized Env (17, 18).

The clade C subtype of HIV-1 is the most prevalent with more than 50% coverage worldwide and remains the major circulating strain in India, Brazil, China, and parts of Africa. We focused our effort on designing an immunogen with our previously identified membrane-bound efficiently cleaved clade C Env, 4-2.J41, of Indian origin. This envelope was of particular interest to us as it binds efficiently to bNAbs but poorly to non-NAbs when expressed on the cell surface (19, 20). Using this Env, we envisaged that by priming with either plasmid DNA or vectors expressing efficiently cleaved membrane-bound Env followed by soluble native-like protein boost, we could improve on the quality and the durability of the immune response in immunized animals (21, 22).

Here, we have stabilized a soluble form of 4-2.J41 Env by swapping the gp41 region from BG505.SOSIP.664 Env into 4–2.J41.SOSIP.664 Env, which otherwise does not form a stable native-like trimer. Size exclusion chromatography (SEC) and negative stain electron microscopy (NS-EM) data have confirmed that the trimers are homogenous, stable, and well ordered displaying a 3-lobed architecture resembling the native HIV-1 spike. Furthermore, biolayer light interferometry (BLI)-based analysis for binding to antibodies have shown that the purified Env binds efficiently to bNAbs and poorly to non-NAbs. Besides, the stability of the trimer at physiological temperature and its high melting temperature makes it a suitable candidate immunogen for testing for prime-boost immunization studies and also for structural analysis.

## Results

**Figure 1.**
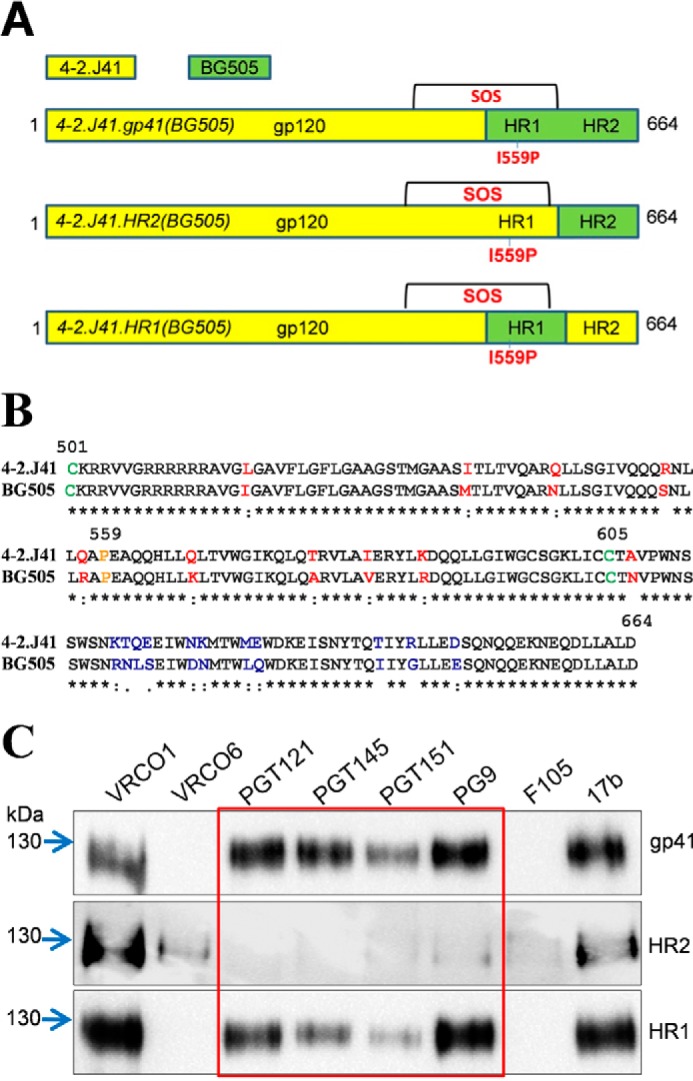
**Design and screening of Envs.** *A,* schematic representation of the various designed Envs. *Yellow* indicates the wild-type 4-2.J41 Clade C Env, whereas *green* is the swapped region from BG505 Env. All constructs bear SOSIP mutations indicated in *red. B,* amino acid sequence alignment in the gp41 region of 4-2.J41 and BG505. Differences are highlighted in *red* (HR1 region) and *blue* (HR2 region). The SOSIP mutations are indicated in *green* and *yellow. C,* screening of designed Envs by IP/Western blot with selected mAbs. Env swapped with full gp41 (4-2.J41.gp41(BG505)) and with the HR1 region (4-2.J41.HR1(BG505)) are recognized by bNAbs (*red box*).

The Indian clade C Env 4-2.J41 is naturally and efficiently cleaved into gp120 and gp41 when expressed on the cell surface and preferentially binds to bNAbs (19). The availability of this Env provided us with the opportunity to modify this protein into a soluble and native-like trimeric form. The introduction of mutations to form 4-2.J41.SOSIP.664 Env failed to produce the desired result. Therefore, we chose to manipulate the N-terminal region of gp41 to test whether this region contributes to the stability of the protein (23). Although high resolution crystal and cryo-EM structures of BG505.SOSIP.664 Env are available, the HR1 region is disordered in most structures (17, 18, 24). The diffused electron density of the HR1 region of BG505 and JRFL Envs in two instances allows to trace the atomic coordinates and build-up the side chain rotamers with some degree of confidence (3, 25). As it was not clear which region of gp41 determines the stability of BG505, we designed three constructs by swapping the DNA sequence coding for the amino acids in gp41 region of BG505 Env to 4-2.J41 Env as described under “Experimental procedures.” The protein forms for the constructs mentioned above were represented as: 4-2.J41.gp41(BG505) Env, 4-2.J41.HR2(BG505) Env, and 4–2.J41.HR1(BG505)Env. Hence, this strategy enabled us to design Envs that displays the clade-specific epitopes in gp120 region of 4-2.J41 Env (Fig. 1*A*). The sequence alignment of the gp41 region between BG505.SOSIP.664 Env and 4-2.J41.SOSIP.664 Env shows that there is a difference of 21 amino acids in that region (Fig. 1*B*).

To screen for the presence of well ordered, native-like trimers, 293F cells were transfected with each of the *env* constructs along with the plasmid expressing cellular protease, furin. The expressed proteins were individually tested for binding to a select panel of bNAbs and non-NAbs. The expressed Envs were immunoprecipitated (IP) followed by Western blot analysis with clade C-specific anti-gp120 antibody (15). The antibodies tested included CD4-bs-directed bNAbs (VRC01 and VRC06), glycan-specific bNAb (PGT121), quaternary conformation dependent bNAb (PGT145), quaternary conformation and cleavage-specific bNAb (PGT151), and quaternary preferring and glycan-dependent bNAb (PG9).

**Figure 2.**
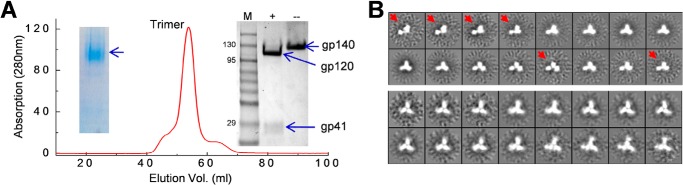
**Size exclusion chromatography and negative-staining electron micrograph.** *A,* SEC profile on a Superdex 200 26/60 column of *G. nivalis* lectin affinity purified 4-2.J41gp41(BG505) shows a single well resolved peak corresponding to trimeric Env. A blue native-PAGE profile is shown in the *right inset*. In the *left inset*, a non-reducing PAGE with (+) and without (−) DTT is shown. The gp140 dissociates into gp120 and gp41 in the presence of DTT. *B,* NS-EM of purified trimeric Env. Reference-free two-dimensional class averages of 4-2.J41.HR1(BG505) (*upper panel*) shows presence of aberrant trimers (indicated with *red arrows*) along with well formed trimers. The 4-2.J41.gp41(BG505) (*lower panel*) shows well formed, compact trimers.

Initial screening showed that the full gp41-swapped Env, 4-2.J41.gp41(BG505), and HR1-swapped Env, 4-2.J41.HR1(BG505), were readily recognized by bNAbs VRC01, PGT121, PG9, PGT145, and PGT151 but not by non-NAbs (Fig. 1*C*). However, the 4-2.J41.HR2(BG505) Env bound to non-NAbs such as F105, b6, A32, C11, and 447–452D (data not shown). It is to be noted that none of the Envs expressed from these constructs recognized the monoclonal antibody, VRC06. This result is in concurrence with a previous study where clade C Env 16055 isolated from a plasma sample of an Indian patient failed to bind VRC06 (26). The antibody directed to CD4-induced (CD4i) conformation, 17b recognized the Envs expressed from all three constructs. The presence of bands corresponding to the Envs in Western blot analysis post-IP with bNAbs that target quaternary structure-specific epitopes of 4-2.J41.gp41(BG505) and 4-2.J41.HR1(BG505) Envs, suggested that the recombinant 4-2.J41.SOSIP.664 Env could be stabilized by swapping either the gp41 subunit or the HR1 region of BG505 Env to 4-2.J41 Env. After the initial screening by IP and Western blot, the 4-2.J41.gp41(BG505) and 4-2.J41.HR1(BG505) Envs were purified by lectin-affinity (*Galanthus nivalis*) column followed by SEC. Blue native-polyacrylamide gel electrophoresis (BN-PAGE) was used to assess the presence of trimeric Env in the purified sample and the quality of the trimer was confirmed by NS-EM. The SEC profile showed a nice single peak, which eluted at ∼55 ml from a Superdex 200 16/60 column for both 4-2.J41.gp41(BG505) and 4-2.J41.HR1(BG505) Envs that corresponds to the elution volume for such trimeric Envs. From the eluent the trimeric Env resolved as a discrete band in BN-PAGE and separated into gp120 and gp41 components in the presence of reducing agent, indicating effective furin cleavage of the SOSIP trimers (Fig. 2*A*). However, under NS-EM, the 4-2.J41.HR1(BG505) Env was found to consist of a heterogeneous mixture of open trimer, some dimers, and closed compact native-like trimers, indicating that the single peak in SEC was associated with aberrant trimers and dimers that could not be resolved by SEC. On the other hand 4-2.J41.gp41(BG505) Env appeared more homogenous with >90% trimers that were 3-lobed propeller-shaped, closed, compact, and native-like as assessed by visual inspection when compared with NS-EM image of BG505.SOSIP.664 Env trimers (Fig. 2*B*). Thus, 4-2.J41.gp41(BG505) Env could be purified to homogeneity by a simple purification protocol, but a more stringent purification protocol was required for 4-2.J41.HR1(BG505) Env involving positive or negative Ab-affinity selection (26). Based on yield and quality of the proteins, we chose to further characterize the 4-2.J41.gp41(BG505) Env.

**Figure 3.**
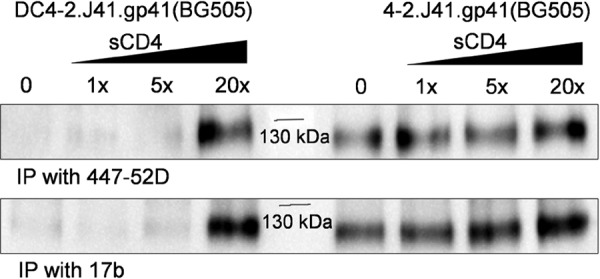
**Conformational change of 4-2.J41.gp41(BG505) and its double cysteine mutant, DC4-2.J41.gp41(BG505) Envs with increased concentration of sCD4.** To check the sCD4-induced conformational changes resulting from the exposure of epitopes for antibodies, 447-52D and 17b, the 4-2.J41.gp41(BG505) Env and the double cysteine mutant (I201C/A433C) Env, DC4-2.J41.gp41(BG505) incubated with increasing molar concentrations of sCD4 (1×, 5×, and 20×) followed by IP/Western blot analysis indicates a 20 m excess of sCD4 is needed to expose epitopes of 447-52D (*top panel*) and 17b (*bottom panel*).

Due to structural flexibility, the soluble form of Env frequently adopts a relatively open conformation exposing epitopes for CD4-induced antibodies leading to a transition from closed “compact” to labile “open” conformation. The CD4 mediates such transition by exposing V3 and bridging sheet epitopes and makes the immunogen less desirable. A double cysteine mutant (I201C/A433C) reported previously prevents CD4 from triggering and preferentially blocks access to CD4-induced non-neutralizing antibody epitopes (18). When the same double cysteine mutant was introduced in 4-2.J41.gp41(BG505) Env, the mutant Env, DC4-2.J41.gp41(BG505), was unable to bind to bridging sheet-directed antibodies, such as 17b and V3 epitope-directed antibody 447-52D. The Env, DC4-2.J41.gp41(BG505) was incubated with an increasing concentration of soluble CD4 (sCD4) followed by IP with either 447-52D or 17b. A 20 m excess of sCD4 was needed to expose the V3 and bridging sheet epitopes in DC4-2.J41.gp41(BG505) Env in comparison to 4-2.J41.gp41(BG505) Env (Fig. 3). The increased band intensity for 4-2.J41.gp41(BG505) Env with increasing concentrations of sCD4 suggested that more and more molecules adopted an open conformation in the presence of sCD4.

**Figure 4.**
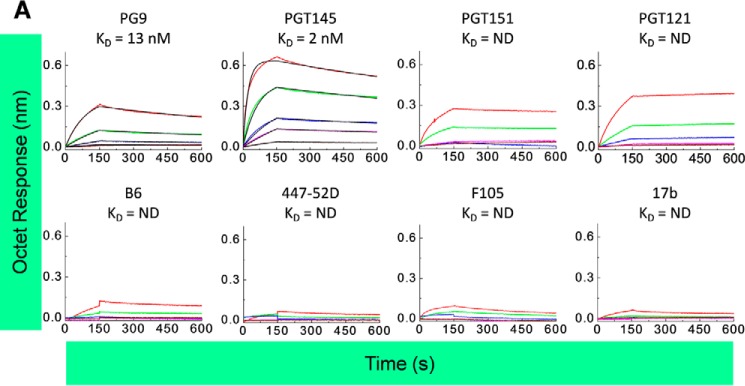
**Antigenic profile of trimeric Env by BLI.** Anti-human Fc sensors were used to capture the mAbs. Trimeric Env DC4-2.J41.gp41(BG505) was used as analyte in various concentrations (210 to 2.6 nm; 1/3rd serial dilution). *Upper panel* represents binding curves obtained with bNAbs, namely PG9, PGT145, PGT151, and PGT121. *Lower panel* represents binding curves with non-NAbs, namely B6, 447-52D, F105, and 17b.

The binding kinetics of the DC4-2.J41.gp41(BG505) Env with antibodies was determined by BLI. Recognition of this Env by bNAbs such as PG9, PGT145, PGT151, and PGT121 that are dependent on quaternary conformation was assessed and the Env bound to all these antibodies with high affinity (Fig. 4, *upper panel*). It is to be noted that the bNAb, PG9 (27), is a conformation-dependent antibody, which asymmetrically interacts with the V1/V2 loop of two protomers of a native trimeric Env. The contact regions primarily include the glycan at Asn-160 and Asn-156 and residue Lys-168 of one protomer and glycans Asn-160 and Asn-197 (V3 loop) of the adjacent protomer (28, 29). Another quaternary-specific, trimer-selective conformational antibody, PGT145 binds to the high mannose glycan-dependent moieties at the trimer apex (V2 loop) (30). As PG9 and PGT145 bind only one site per trimer and are not subject to avidity gain, it helped us to calculate the affinities for these trimer-preferring bNAbs after performing binding kinetics by BLI. The calculated affinity for PG9 and PGT145 are in the nanomolar range (*K_D_* for PG9, 13 nm; *K_D_* for PGT145, 2 nm). These results suggested that the designed envelope is trimeric and native-like in conformation with appropriately placed glycans at positions Asn-160, Asn-156, and Asn-173 that are hallmarks of nanomolar affinity to PG9 and PGT145. The trimer and cleavage-specific bNAb PGT151 (31) recognizes a conformational epitope present at the interface of gp120 and gp41 that is better exposed in cleaved trimers (32) and binds with an estimated stoichiometry of two antibody molecules per trimer (33). The DC4-2.J41.gp41(BG505) Env trimer bound strongly to PGT151 with a fast on-rate, which was comparable with PG9. A very slow dissociation curve suggested that the purified Env not only forms native-like trimers but also was properly cleaved and maintained quaternary structure. The bNAb, PGT121 that recognizes the Asn-332 supersite bound with a fast on-rate but with no dissociation, and in fact secondary association was seen during wash, indicating very tight binding. Therefore the dissociation constant could not be calculated.

Another critical feature of a native-like Env is not to expose the epitopes for non-NAbs (33). To investigate whether DC4-2.J41.gp41(BG505) could bind to non-NAbs, binding kinetic studies with CD4b-directed, F105 and b6, V3-directed antibody, 447-52D, and bridging sheet epitope-directed 17b were performed and the binding to those non-NAbs was found to be weak (Fig. 4, *lower panel*). In some cases, the binding affinity was roughly calculated based on only the highest concentration of the analyte indicating binding in the micromolar range, suggesting that this trimeric Env did not expose the epitopes for non-neutralizing antibodies.

**Figure 5.**
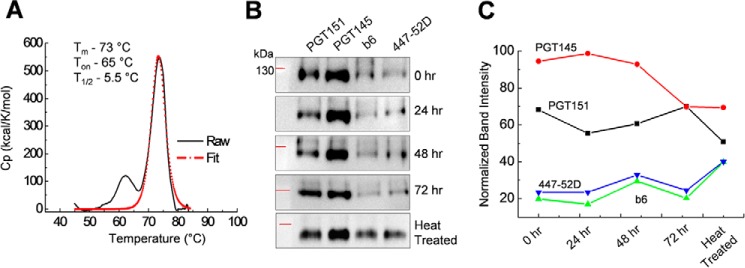
**Thermostability of DC4-2.J41.gp41(BG505) trimer.** *A,* DSC analysis of the purified DC4-2.J41.gp41(BG505) trimer reveals a melting profile with *T_m_* value of 73 °C. *B*, IP/Western blot profile of DC4-2.J41.gp41(BG505) with bNAbs (PGT151 and PGT145) and non-NAbs (b6 and 447-52D) after incubation at 37 °C for different time points. *C,* intensity plot using ImageJ of bands obtained after IP/Western blot analysis in Fig. 5*B*, showing time-dependent changes in bNAbs and non-NAbs binding.

Next, we tested the thermostability of the designed trimeric Env by measuring the melting temperature (*T_m_*) using differential scanning calorimetry (DSC). The *T_m_* for DC4-2.J41.gp41(BG505) Env was ∼73 °C with an onset of melting at ∼65 °C (Fig. 5*A*). The high *T_m_* obtained for the DC4-2.J41.gp41(BG505) Env was comparable with the double cysteine mutant BG505.SOSIP.664 Env (18). Another important parameter in terms of stability is the prolonged survival of function at physiological temperature (37 °C). To measure the same, the trimer was incubated at 37 °C for different time periods ranging from 24 to 72 h. One fraction was incubated at 75 °C for 5 min. These fractions were then immunoprecipitated with either bNAbs (PGT145 and PGT151) or non-NAbs (b6 and 447-52D) followed by Western blot analysis (Fig. 5*B*). The intensity of the band obtained after Western blot analysis was quantified using NIH ImageJ software (34). The data clearly showed that after 72 h of incubation at 37 °C, only a minimal decrease in binding to bNAbs was observed and an increase in binding corresponding to non-NAbs was observed. Even when heated at 75 °C for 5 min, a fraction of the population maintained the native-like trimeric conformation that bound to bNAbs (Fig. 5*C*). The maintenance of native conformation after prolonged incubation at 37 °C indicated that this Env was stable at physiological temperature.

## Discussion

To elicit neutralizing antibodies, a trimeric Env immunogen in soluble pre-fusion form that mimics the native quaternary conformation of Env present on the viral surface is preferred. The SOSIP mutations along with truncation before the membrane proximal external region at amino acid 664 in BG505 Env stabilizes the protein in the pre-fusion state (35) and has been found to be critical for the production of high-quality trimers. The SOSIP.664 trimeric form of clade A Env BG505, and clade B Env B41, show almost similar antigenic profiles corresponding to Env spikes on the viral surface (13, 36). Two other SOSIP.664 forms of trimeric clade C Envs, DU422 and ZM197M, have also been purified and characterized. The antigenicity and other biophysical parameters have been shown to be comparable with Env present on the viral surface (37). However, it is not clear whether these Envs in their wild-type (WT) full-length form are efficiently cleaved when they are expressed on the cell surface as the uncleaved or partially cleaved Envs do not display native-like trimeric forms when expressed as membrane-bound forms on cell surface. So, the advantages of soluble native-like trimeric, Envs designed from efficiently cleaved membrane-bound full-length Envs on the cell surface are 2-fold: 1) the native-like Envs can be compared with the functional native Env expressed on the cell surface; 2) it can be used for priming with either plasmid DNA or vector or virus-like particle (VLP) followed by native-like trimeric protein boost for immunization. Additionally, the extensive sequence diversity and labile nature of the Env spike led us to go forward with the designing of a soluble native-like trimeric HIV-1 clade C Env of Indian origin, which is efficiently cleaved on the cell surface. However, the purified SOSIP.664 form of Env, 4-2.J41, was found to be a heterogeneous mixture of non-native-like trimeric population. It has been shown that the non-native forms of trimers and other Env contaminants can be removed by specific antibody selection (26, 36). Unfortunately, by using the purification methods described above, including positive and negative selection, we were not successful in getting the native-like trimeric form of 4-2.J41.SOSIP.664 Env.

It has been described elsewhere how JRFL, a membrane-bound efficiently cleaved clade B Env has been stabilized by swapping the HR1 of gp41 region of another relatively more stable Env, KNH1144 from clade A (23). In another similar study, the trimeric state of HIV Env, JRFL, has been stabilized through modification of the N-terminal region of gp41 with the help of Simian immunodeficiency virus Env sequence (38). However, binding to non-neutralizing antibodies to those Envs have not been demonstrated. It is to be noted that native Env does not bind to non-neutralizing antibodies (33). It is also unclear whether the above engineered Envs maintains its native conformation as the antibody-Env binding kinetics using native conformation-dependent antibodies and EM studies of the proteins have not been described. So, we considered substituting either HR1 or HR2 or both of gp41 from BG505 Env to 4-2.J41 Env, as it is relatively more stable in its SOSIP.664 form. Our data generated from the swapping experiments indicate that gp41 contributes to instability of 4-2.J41 Env. To map the domain required for stabilization of wild-type 4-2.J41.SOSIP.664, several mutations were introduced by comparing the sequence with BG505. However, this did not bring a change in the result (data not shown). Furthermore, a homogenous stable conformation of 4-2.J41.SOSIP.664 could not be obtained by replacing the HR1 of gp41 where there is a difference of 10 amino acids (Fig. 1*B*) between BG505 and 4-2.J41 Envs. The complete swapping of gp41 from BG505.SOSIP.664 to 4-2.J41 Env where there is a difference of 21 amino acids made the protein homogenously stable in a native-like trimeric form. Furthermore, the 4-2.J41.gp41(BG505) Env can be purified by a lectin affinity column followed by SEC and did not require elaborate positive or negative selection by specific mAbs. Under NS-EM the purified protein showed >90% trimer with a closed compact structure when compared visually with NS-EM of BG505.SOSIP.664 Env. Stabilizing the bridging sheet locking double cysteine (I201C/A433C) mutations further stabilized the trimers in a closed compact conformation that needed ×20 m excess of sCD4 to expose the V3 and bridging sheet-dependent non-neutralizing epitopes compared with unmutated trimeric Env.

An important parameter for well formed native-like, closed compact trimers is its ability to bind to bNAbs without binding to non-NAbs. This feature is a prerequisite for a candidate immunogen as they are expected to expose only the epitopes for bNAbs when administered to the host. When our designed construct expressed proteins, and were analyzed for binding against a panel of antibodies both broadly and non-neutralizing by BLI, it was found to specifically bind to bNAbs with nanomolar affinity but the affinity for non-NAbs was ∼1000-fold less. Another desired feature of an immunogen is its ability to maintain a closed, native-like, trimeric quaternary conformation when exposed to physiological temperature after injection into a host. The designed Env immunogen maintained desirable conformation and retained its ability to bind to bNAbs even after 3 days of incubation at 37 °C. Furthermore, DC4-2.J41.gp41(BG505) Env trimers when assessed by DSC presented an appropriate stability parameter with melting temperature of ∼73 °C with the onset of melting at ∼65 °C. Studies have shown that there is consistent autologous neutralization with increased stability of the native-like trimeric Env due to increased resistance to unfolding and it suggests that this parameter should be considered for immunogen design (39). The highly stable characteristics displayed by trimeric Envs might help in increasing the number of native-like Env in pseudo-typed VLPs, which might make VLPs more effective as immunogens by enhancing the avidity of antibody to Env. Moreover, with high thermal stability, this trimeric Env might maintain native state and continuously present neutralizing epitopes to B cells. Most importantly, in this study, we have stabilized the soluble form of 4-2.J41, a clade C HIV-1 Env of Indian origin, obtained after screening a panel of Envs for its efficient cleavage on the cell surface and the selection of this Env will provide an opportunity to prime with either plasmid DNA, or by viral vector or even VLPs.

In summary, here, we have taken a novel approach to stabilize 4-2.J41 Env, which was otherwise difficult using strategies described earlier. HIV-1 displays unprecedented enormous diversity of virus circulating in human population across the globe. As Env is the main target of neutralizing antibodies, Env-based vaccine candidates addressing this diversity are essential. The approach used in this study could be applicable to other Envs of diverse clades to generate a repertoire of highly stable native-like trimers.

## Experimental procedures

### Construct design, protein expression, and purification

Three constructs were designed by swapping either the DNA sequence coding for amino acids in the gp41 region of BG505 *env* to 4-2.J41 *env* (4-2.J41.gp41 (BG505) *env*) or only the amino acids in the HR2 region (4-2.J41.HR2(BG505) *env*) or the amino acids in the HR1 region (4-2.J41.HR1(BG505) *env*). All *envs* terminated at 664 amino acids. Furthermore, in all constructs mutations were introduced to, including SOSIP and the six arginine (6R) residues at the furin cleavage site, all the constructs were prepared from codon-optimized 4-2.J41 (Clade C) by using PCR to swap regions of interests with BG505 and cloned in pcDNA3.1(+) vector. For all constructs, we used the CD5 leader sequence at the N-terminal to enhance protein expression. Envs were expressed in transiently transfected 293F cells in suspension culture using 293fectin (Invitrogen) with the *env* of interest and plasmid carrying the *furin* gene added in a 2:1 ratio for maximize cleavage. The protein forms for the constructs mentioned above were represented as 4-2.J41.gp41(BG505) Env, 4-2.J41.HR2(BG505) Env, and 4-2.J41.HR1(BG505) Env. Cell culture supernatants were harvested 5–6 days after transfection. For protein purification the supernatant was passed through a *G. nivalis* lectin-agarose (Sigma) column. Bound proteins were eluted with HEPES buffer (50 mm HEPES, 75 mm NaCl, pH 7.5) containing 1 m methyl α-d-mannopyranoside. Eluted proteins were dialyzed against HEPES buffer, concentrated with an Amicon filter (Millipore; 100 kDa), and loaded on a HiLoad Superdex 200 (16/60) column (GE Biosciences) in the HEPES buffer background. Trimer fractions were collected and confirmed by Blue Native-PAGE (4–12% gradient; Bio-Rad) analysis. Pooled trimeric fractions were aliquoted and stored in −80 °C until further use.

### Immunoprecipitation

For IP, 2 ml of crude supernatant or 1 μg of purified protein was incubated overnight at 4 °C with 2 μg of mAbs of choice in the presence of protein G-agarose (100 μl of 50% slurry; G Biosciences) followed by washing of beads with PBS before SDS-PAGE analysis.

### PAGE and Western blot analysis

For Western blot analysis, protein was transferred from PAGE to PVDF membrane. The membrane was blocked with 5% skimmed milk, incubated with primary Ab, anti-gp120 Env (clade C) (Abl Inc.), 1:500 dilution overnight at 4 °C. The membrane was developed with HRP-conjugated anti-rabbit secondary Ab (Thermo).

### Bio-layer interferometry (Octet Red)

For binding kinetics anti-human Fc sensors (ForteBio Inc.) was used to capture the mAbs and trimer was used as analyte in various concentrations (210 to 2.6 nm; 1/3rd serial dilution) in the HEPES buffer background supplemented with 0.02% Tween 20 and 0.1% BSA. The experiment was performed at room temperature with agitation at 1000 rpm. To capture the antibodies the biosensors were immersed in wells containing mAbs at a concentration of 10 μg/ml for 120 s. Association was recorded for 150 s followed by dissociation for 450 s. Data were analyzed using the ForteBio Data Analysis software, 9.0 (ForteBio Inc). The kinetic parameters were calculated using a global fit 1:1 model applicable for mAbs.

### Negative stain electron microscopy

Trimer samples were diluted to ∼0.02 mg/ml in Tris-buffered saline and applied to a carbon-coated 400 copper mesh grid. Further data collection and analysis procedures have been previously described (40).

### Differential scanning calorimetry

Thermal melting was analyzed with a Nano-DSC (TA instruments). Samples were dialyzed in HEPES buffer (50 mm HEPES, 75 mm NaCl, pH 7.5) and protein concentration was adjusted to 0.7 mg/ml prior to measurement. Thermal melting was performed at a scanning rate of 1 °C/min under 3.0 atmospheres of pressure. Data collected were analyzed with NanoAnalyze software, 3.6.0 (TA instruments), after buffer correction, normalization, and baseline subtraction.

## Author contributions

S. A. and B. K. C. designed the immunogens; S. A., T. S., and N. K. carried out immunogen expression, biochemical, and biophysical characterization. G. O. and A. W. carried out EM data acquisition and analysis. S. A. and B. K. C. analyzed the data and wrote the original draft; T. S., G. O., and A. W. edited the draft.

## Footnotes

3 The abbreviations used are:

Env: envelope glycoprotein
bNAb: broadly neutralizing antibody
SEC: Size exclusion chromatography
NS-EM: negative stain electron microscopy
BLI: biolayer light interferometry
DSC: differential scanning calorimetry
gp: glycoprotein
IP: immunoprecipitation
VLP: virus-like particle.
